# Interaction of Sodium Polystyrenesulfonate with Fluorinated Ionic Surfactant of Opposite Charge

**DOI:** 10.1021/acs.langmuir.5c02918

**Published:** 2025-09-22

**Authors:** Matevž Turk, Ksenija Kogej, Per Hansson

**Affiliations:** † Department of Physical Chemistry, Faculty of Chemistry and Chemical Technology, 37663 University of Ljubljana, Vecna pot 113, 1000 Ljubljana, Slovenia; ‡ Department of Medicinal Chemistry, 8097 Uppsala University, Box 574, SE-75123 Uppsala, Sweden

## Abstract

Polyelectrolyte–surfactant systems of opposite charge have been widely studied because of their relevance in applications ranging from pharmaceuticals to advanced materials. However, the role of hydrophobic interactions in such systems remains debated, particularly for sodium poly­(styrenesulfonate) (NaPSS) and cationic surfactants. This study investigates the binding behavior of NaPSS with the conventional hydrocarbon surfactant dodecylpyridinium chloride (DPC) or the fluorinated surfactant 1*H*,1*H*,2*H*,2*H*-perfluorodecylpyridinium chloride (HFDePC), with the objective of elucidating the importance of hydrophobic interactions in such systems. The binding isotherms of both surfactants were measured in both linear NaPSS solutions and covalently cross-linked NaPSS hydrogels. HFDePC exhibited a binding isotherm with a negative slope, indicative of unique binding behavior. Thermodynamic modeling revealed that the negative slope arises from the formation of metastable colloidal states. For DPC, modeling indicated the formation of mixed micelles with NaPSS with a consistent surfactant/polyion charge ratio, explaining its lower cooperativity and atypical phase behavior. In NaPSS hydrogels, the swelling isotherms revealed a uniform hydrogel collapse in the case of DPC and the formation of biphasic core–shell structures in the case of HFDePC. Small-angle X-ray scattering showed rod-like micelle formation for both systems, with increased micelle length at higher binding ratios and the emergence of hexagonal packing of micelles near coil saturation. Overall, the findings of this study underscore the importance of surfactant and polyelectrolyte structure and hydrophobicity and offer new insights into the nature of interactions in polyelectrolyte–surfactant systems of opposite charge.

## Graphical abstract

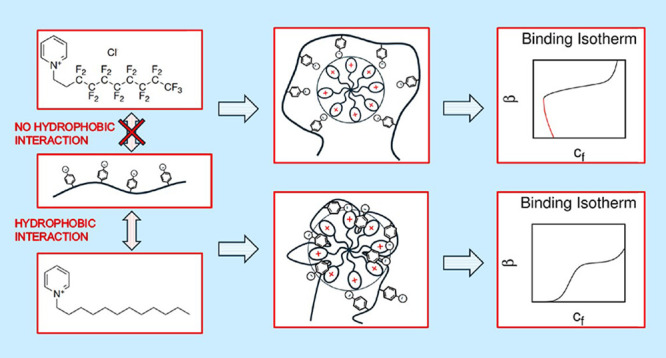

## Introduction

Polymer–surfactant systems are an active area of research because of their broad range of technological applications. Their utility spans from industrial uses, such as detergents and paints,^1,2^ to pharmaceutical and biomedical products.^3–9^ Among these, polyelectrolyte–surfactant systems of opposite charge have attracted particular interest as promising candidates for drug delivery applications.^10–13^ When considering a polyelectrolyte–surfactant ion pair for applications, special attention must be given to the interactions within the system, as these can alter the properties of individual components and determine the system’s overall behavior. Understanding these interactions is therefore crucial for effectively harnessing the utility of a given polyelectrolyte–surfactant mixture.

Polyelectrolytes and ionic surfactants of opposite charge, when present together in solution, form supramolecular complexes.^3^ This association is a highly cooperative process and begins at a well-defined surfactant concentration, commonly referred to as the critical association concentration (CAC).^3–5,9^ A standard way to track polyion–surfactant association is through surfactant binding isotherms,^3,4,9^ which are constructed by plotting the fraction of surfactant molecules in PE-surfactant complexes against the fraction of surfactant molecules that remain free in solution. The fraction of complexated surfactant molecules is usually expressed as the degree of binding, β, which is defined as the mole amount of bound surfactant molecules, *n*_b_, normalized to the mole amount of monomer units of the polyelectrolyte, *n*_m_, as given by eq 1.

$$\beta =\frac{n_{b}}{n_{m}}$$ 1

The fraction of the free surfactant molecules is expressed simply as the log value of the free surfactant concentration, i.e., log *c*_f_. A typical surfactant binding isotherm has a sigmoidal shape, with three distinct regions: a low binding region at low surfactant concentrations, a sharp increase in binding once the CAC is reached, and a plateau at high surfactant concentrations where the polyelectrolyte becomes saturated with surfactant molecules.^3,4,9^

Fluorinated surfactants have been receiving increasing attention due to their unique properties, including low surface tension and high thermal and chemical stability.^14–16^ These characteristics make them promising candidates for specialized applications, ranging from inert media to electrolytes in solar cells and batteries.^17–20^ While numerous studies have explored ionic fluorinated surfactants, their interactions with oppositely charged polyelectrolytes in solution remain unexplored. This study aims to address this gap and present the first insights into these interactions.

The major research question that we address is the role of the hydrophobic interaction between polyelectrolytes and surfactants of opposite charge. This is related to a recent controversy in the literature on the properties of complexes formed between sodium poly­(styrenesulfonate) (NaPSS) and cationic surfactants in bulk solutions and at the air/water interface.^21–24^ In particular, there are opposing opinions on whether the phase behavior of such systems differs from that of other oppositely charged polyelectrolyte–surfactant systems. The low cooperativity of binding of cationic surfactants to NaPSS in comparison to that of other polyelectrolyte–surfactant systems has been an important part of the argumentation. Unfortunately, the importance of the hydrophobic nature of the NaPSS chain’s backbone has been left out of the discussion, although earlier work has demonstrated its importance.^25–27^ Fluorinated surfactants are interesting in this respect because hydrocarbons and fluorocarbons do not mix readily. As a result, the binding of fluorinated surfactants to NaPSS is expected to be minimally influenced by hydrophobic interactions and instead resemble systems where binding is governed primarily by electrostatic interactions.

This work features a comparative study of systems composed of either the conventional hydrocarbon surfactant dodecylpyridinium chloride (DPC) or the fluorinated surfactant 1*H*,1*H*,2*H*,2*H*-perfluorodecylpyridinium chloride (HFDePC) in combination with NaPSS. The chemical structures of DPC, HFDePC, and NaPSS are shown in Figure 1. Binding isotherms of both surfactants were first determined to linear NaPSS in solution via potentiometric titration method using a surfactant ion-selective electrode (SSE) and subsequently to covalently cross-linked NaPSS hydrogels. The hydrogel samples with bound surfactant were further analyzed using small-angle X-ray scattering (SAXS) to gain structural insights into the polyelectrolyte–surfactant aggregates formed within them. The experimental data were then used to parametrize two theoretical models—one for the NaPSS-DPC system and another for the NaPSS-HFDePC system—allowing for the calculation of theoretical binding isotherms and an investigation of the interplay between different types of interactions in these systems.

**Figure 1:**
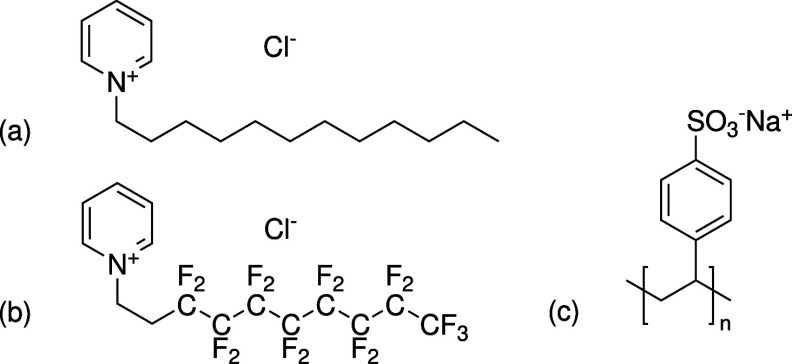
Chemical structures of (a) DPC, (b) HFDePC, and (c) NaPSS.

## Materials and Methods

### Materials

The 1*H*,1*H*,2*H*,2*H*-perfluorodecylpyridinium chloride was obtained as a gift from Professor Asakawa of the University in Kanazawa, Japan. The detailed synthesis of HFDePC is described in their work.^28^ Analytical grade sodium chloride was dried at 110 °C and stored in the desiccator. NaPSS with a molar mass of about 70,000 Da and a degree of sulfonation of 1.0, supplied by Polysciences, Inc., was prepared and purified according to a procedure described in the literature.^29–31^ All other reagents were obtained from commercial suppliers and were used without further purification.

### Experimental Methods

#### Potentiometric Titration Using Surfactant Ion-Selective Electrode

Potentiometric titration using the surfactant ion-selective electrode (SSE) was used to determine binding isotherms of DPC, HFDePC, and their mixtures, to linear NaPSS in aqueous solution. The SSE was prepared following a modified procedure adapted from the literature,^32–34^ described in detail in Section S1.1 of the Supporting Information (SI). The saturated calomel electrode (SCE) was used as the reference electrode. The experimental setup for potentiometric titration consisted of a titration chamber equipped with the SSE, a micro buret filled with the titrant surfactant solution, and a stir bar. Calibration measurements were performed by recording the potential difference between the SSE and SCE after each addition of the surfactant titrant solution into 10 mL of solvent (aqueous NaCl or deionized water) using the Iskra MA 5740 pH meter. The stability criterion for taking a reading after each addition was d*E*/d*t* = 0.1 mV/60 s. The solution was continuously stirred and a constant temperature of 25 °C was maintained during the titration. Titrations for the binding isotherm determination were performed in the same manner as described above. Instead of aqueous NaCl or water, the titration chamber was filled with 10 mL of 1 × 10^–4^ M NaPSS solution in the selected solvent (aqueous NaCl or deionized water), to which surfactant titrant solution was gradually added. The stability criterion for taking a reading after each addition was d*E*/d*t* = 0.1 mV/180 s. The degree of surfactant binding to NaPSS, β, was calculated as given by eq 2:

$$\beta =\frac{c_{t}-c_{f}}{c_{p}}$$ 2

where *c*_t_ and *c*_f_ represent the total and free surfactant concentrations, respectively, which are obtained from the potentiometric curves measured in the absence and presence of NaPSS, and *c*_p_ is the concentration of the PSS polyion expressed in moles of monomer units per unit volume.

#### Determination of Surfactant Binding Isotherms to NaPSS Hydrogels

Determination of the binding isotherms of DPC and HFDePC to NaPSS in the form of a cross-linked hydrogel was done by equilibrating the hydrogel pieces under surfactant solutions of various concentrations. NaPSS hydrogels were synthesized following a modified procedure adapted from the literature,^27,35,36^ described in detail in Section S1.2 of the Supporting Information. Three sets of 10 mL aqueous solutions were prepared, containing either DPC or HFDePC. These solutions spanned a range of total surfactant concentrations from 0.15 to 3.6 mM. A NaPSS hydrogel piece, weighing approximately 1 g, was added to each solution. The systems were allowed to equilibrate for 2 weeks. After equilibration, the free surfactant concentration in the solutions above the hydrogels was determined by means of potentiometry and UV–vis spectroscopy. Potentiometric measurements were performed with the SSE and interpreted using the calibration curves determined as described above. Spectroscopic measurements were performed on the Cary 100 Bio UV–vis spectrometer. The measurements were done at 260 nm, near the peak in the extinction coefficient value for both DPC and HFDePC. Using the exact initial hydrogel masses, *m*_0_, and assuming the hydrogel density of ρ_gel_ = 1 g/mL, the β values were calculated using eq 3:

$$\beta =\frac{V_{\mathrm{aq}}\rho_{\mathrm{gel}}(c_{t}-c_{f})}{c_{m,\mathrm{gel}}m_{0}}$$ 3

where *V*_aq_ represents the total volume of the system and *c*_m,gel_ = 27 mM is the monomer concentration of NaPSS within the hydrogels in their initial state (see Section S1.2 of the SI).

The equilibrated hydrogel pieces were weighed, and swelling isotherms were constructed by plotting the ratio of the equilibrium and the initial hydrogel masses against the corresponding β values. Additionally, the hydrogel pieces were analyzed using SAXS. The gel pieces were cut with a scalpel and mounted in a special vacuum sealed sample holder for gels/pastes/viscous samples from Xenocs. The gel pieces were sandwiched between two Kapton windows; sample thickness: 1.0 mm. The SAXS measurements were performed on Xeuss 2.0, Xenocs X-ray system equipped with a GeniX 3D microfocus X-ray source and Pilatus3 R 300 K detector operating without a beam stop. Selected samples of equilibrated gels were recorded for 30 min at a 2000 mm sample-to-detector distance. The scattering curves were preprocessed with Xenox XSACT 2.4 software^37^ and analyzed with SasView 6.0 software.^38^

### Theoretical Models

The values of parameters appearing in our models were either based on our experimental results or adopted from the literature.^9,27,39–49^ Detailed description and parametrization of both models, as well as the binding isotherm calculation procedures, are provided in Section S2 of the Supporting Information.

#### Model of the NaPSS-DPC System

In the NaPSS-DPC system, the surfactant ions (DP^+^) and the styrenesulfonate segments of the PSS chain (SS^–^) are modeled to form mixed micelles. This was motivated by studies showing that polyelectrolytes featuring hydrophobic functional groups on their repeating units (such as PSS) tend to penetrate the surfactant micelles due to the hydrophobic effect.^50^ For example, Gao et al. have determined that styrene groups of the PSS chains are located near the β-CH_2_ groups of surfactant molecules in micelles using NMR.^25^ A mixed micelle consists of a water-free core composed of the hydrocarbon tails of DP^+^ and the hydrophobic parts of the SS^–^ segments, and a charged interface formed by the positively charged pyridinium groups of the surfactant and the negatively charged sulfonate groups of the polyelectrolyte. By choosing a micelle composition, the chemical potentials of DP^+^ and SS^–^ in the micelle subphase, μ_*i*_^mic^, can be calculated as given by eq 4:

$$\mu_{i}^{\mathrm{mic}}=\mu_{i}^{0,\mathrm{mic}}+\mu_{i}^{\mathrm{surf},\mathrm{mic}}+\mu_{i}^{\mathrm{el},\mathrm{mic}}+\mu_{i}^{\mathrm{mix},\mathrm{mic}}$$ 4

In eq 4, the terms on the right-hand side represent the standard chemical potential of species *i* in the micelle subphase, the surface free energy contribution, the electrostatic free energy contribution, and the entropy of mixing contribution to the chemical potential of *i*, respectively. Additionally, the chemical potentials of DP^+^ and SS^–^ in the solution surrounding the micelles, μ_*i*_^w^, can be expressed as given by eqs 5 and 6:

$$\mu_{{\mathrm{DP}}^{+}}^{w}=\mu_{{\mathrm{DP}}^{+}}^{0,w}+k_{B}T\ln\left(\frac{c_{{\mathrm{DP}}^{+}}}{c_{0}}\right)$$ 5

$$\mu_{{\mathrm{SS}}^{-}}^{w}=\mu_{{\mathrm{SS}}^{-}}^{0,w}+\mu_{{\mathrm{SS}}^{-}}^{\mathrm{el},w}$$ 6

At equilibrium, the chemical potential of the *i*-th species in the micelle subphase is equal to its chemical potential in the solution (eq 7):

$$\mu_{i}^{\mathrm{mic}}=\mu_{i}^{w}$$ 7

Using this constraint, along with the mass balance and the electroneutrality condition, the free surfactant concentration and the degree of surfactant binding β can be calculated for a given micelle composition. By repeating the calculation for different micelle compositions, the binding isotherm is obtained.

#### Model of the NaPSS-HFDePC System

In the NaPSS-HFDePC system, the interactions between polyelectrolytes and surfactants are predominantly electrostatic. These interactions closely resemble those in systems composed of conventional hydrocarbon ionic surfactants and oppositely charged hydrophilic polyelectrolytes, such as sodium polyacrylate.^3,4,9,51^ Such systems can be modeled as a dilute solution of single polyelectrolyte coils within which surfactant micelles, surfactant unimers, simple salt ions, and water can be enclosed.^52^ Each individual coil, along with all the enclosed species, is treated as a small thermodynamic system in equilibrium with the bulk solution, which contains surfactant unimers and simple salt ions but no micelles. The free energy expression of a polyelectrolyte coil system can be expressed as a sum of separable contributions as given by eq 8:

$$A=A^{\mathrm{el}}+A^{\mathrm{mix}}+A^{\mathrm{trans}}+A^{\mathrm{surf}}+A^{\mathrm{def}}$$ 8

The terms on the right-hand side of eq 8 represent the electrostatic free energy, the free energy of mixing, the free energy associated with transferring fluorocarbon tails from an aqueous environment to the micelle core, the surface free energy, and the configurational free energy of the polyelectrolyte coil, respectively. The chemical potential of the *i*-th species within the polyelectrolyte coil system, μ_*i*_^coil^, can be obtained by taking the partial derivative of eq 8 with respect to the number of *i* molecules, *N*_*i*_ (eq 9):

$$\mu_{i}^{\mathrm{coil}}={\left(\frac{\partial A}{\partial N_{i}}\right)}_{T,V,N_{j}}$$ 9

where *i* can represent water molecules, surfactant unimers, or simple salt ions. The chemical potential of these species in the bulk solution can be expressed as given by eq 10:

$$\mu_{i}^{\mathrm{bulk}}=k_{B}T\ln\left(\frac{c_{i}^{\mathrm{bulk}}}{c_{0}}\right)$$ 10

where *c*_*i*_^bulk^ is the bulk concentration of the *i*-th species and *c*_0_ is the concentration of water (55.5 M). At equilibrium, the chemical potential of each electroneutral species in the polyelectrolyte coil phase is equal to its chemical potential in the bulk solution (eq 11):

$$\mu_{i}^{\mathrm{coil}}=\mu_{i}^{\mathrm{bulk}}$$ 11

Additionally, the equilibrium between surfactant unimers and surfactant micelles must be considered within polyelectrolyte coil systems (eq 12):

$$\mu_{S^{+},\mathrm{mic}}^{\mathrm{coil}}=\mu_{S^{+}}^{\mathrm{coil}}$$ 12

where μ_S^+^,mic_^coil^ represents the chemical potential of surfactant molecules in the micelles within the polyelectrolyte coils and is obtained by taking the partial derivative of eq 8 with respect to the number of surfactant molecules in the micelles. Using these constraints, along with the mass balance and the electroneutrality condition, the binding isotherm can be calculated.

## Results and Discussion

### Surfactant Binding to Linear NaPSS in Solution

First, we determined the binding isotherms of pure DPC, pure HFDePC, and the 3:1, 1:1, and 1:3 DPC/HFDePC mixtures to linear NaPSS in 0.01 M NaCl using the potentiometric titration method described in section Materials and Methods. The results of these measurements are presented in Figure 2 (left). Among all the tested cases, the binding isotherm of pure HFDePC displayed the lowest CAC value, indicating that the interactions between PSS and the surfactant molecules are strongest in this case. Interestingly, the CAC values of all tested DPC/HFDePC mixtures have turned out to be very similar to that of the pure DPC. Moreover, the shapes of the DPC/HFDePC mixture binding isotherms closely resemble that of pure DPC, showing that in mixtures of the two surfactants, DPC dictates the binding regime.

**Figure 2:**
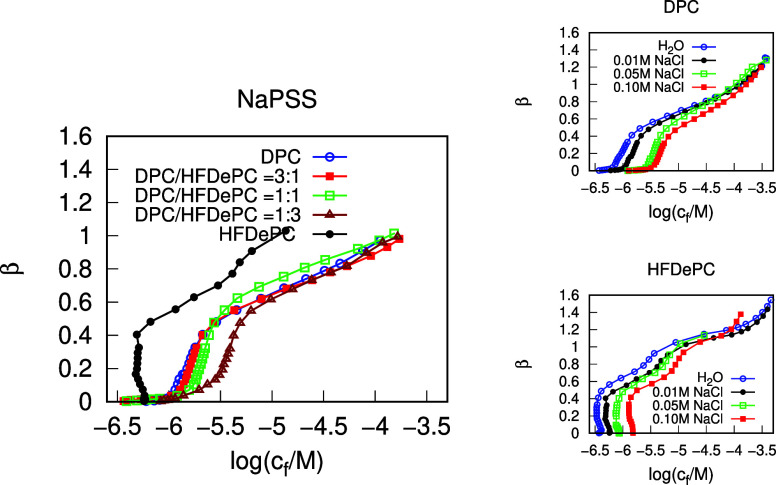
Binding isotherms of DPC, HFDePC, and the 3:1, 1:1, and 1:3 DPC/HFDePC mixtures to linear NaPSS in 0.01 M NaCl, determined by potentiometric titration (left). Binding isotherms of DPC and HFDePC to linear NaPSS, determined by potentiometric titration at various NaCl concentrations (right).

While the binding isotherms of pure DPC and all mixtures follow the “ordinary” shape, the binding isotherm of pure HFDePC displays a negative slope in the cooperative binding region, a rather unexpected feature. To the best of our knowledge, a binding isotherm with negative slope has only been reported for dodecyltrimethylammonium bromide in solutions of sodium hyaluronate in pure D_2_O.^53^ However, in that system, a fraction of the bromide ions was shown to remain bound to the polyelectrolyte-bound micelles, and so the effect resembled that of ionic surfactants above the cmc in pure water.^54^ In contrast, the negative slope in the present case is observed in solutions containing excess of added salt, where the electrostatic effect responsible for the negative slope in polyelectrolyte-free solutions is removed.

Furthermore, we examined the dependency of the DPC and HFDePC binding isotherms on the ionic strength of the solution. Previous reports in the literature suggest that with increasing ionic strength, surfactant binding isotherms tend to shift toward higher free surfactant concentrations, as the addition of simple salt ions screens electrostatic interactions between the surfactant ions and the polyelectrolyte.^4,8,9^ The binding isotherms of DPC and HFDePC to linear NaPSS at various NaCl concentrations are presented in Figure 2 (right). In both the DPC and HFDePC cases, the expected trend is observed. Notably, the negative slope of the HFDePC binding isotherm remains unaffected by the addition of simple salt.

From this point on, we shifted our objective toward explaining the negative slope observed in the HFDePC binding isotherm, as understanding this phenomenon would provide valuable insight into the nature of interactions in the fluorinated surfactant - polyelectrolyte systems.

### Surfactant Binding to NaPSS Hydrogels

There is reason to believe that, due to its hydrophobic nature, PSS may interact with the PVC ion-selective membrane of the SSE, potentially interfering with the potentiometric measurements of free surfactant concentration. Evidence for this possibility comes from the observation that in NaPSS solutions without surfactant, a potential shift of up to −80 mV was recorded in the presence of 0.01 M NaCl relative to the polyelectrolyte-free medium, with an even more pronounced shift observed in the absence of salt. In light of this, our initial concern was to examine whether the negative slope of the HFDePC binding isotherm results from the interference between PSS and SSE. To achieve this, the binding of DPC and HFDePC to covalently cross-linked NaPSS hydrogel networks instead of to linear NaPSS in solution was followed. This approach ensured that, when conducting potentiometric measurements, no polyelectrolyte would be present in the solution in contact with the SSE. Additionally, the free surfactant concentration in the test solutions could be monitored by means of UV–vis spectroscopy, providing another independent determination of the free surfactant-ion concentration to validate our results. The binding isotherms of DPC and HFDePC to NaPSS hydrogels are presented in Figure 3.

**Figure 3:**
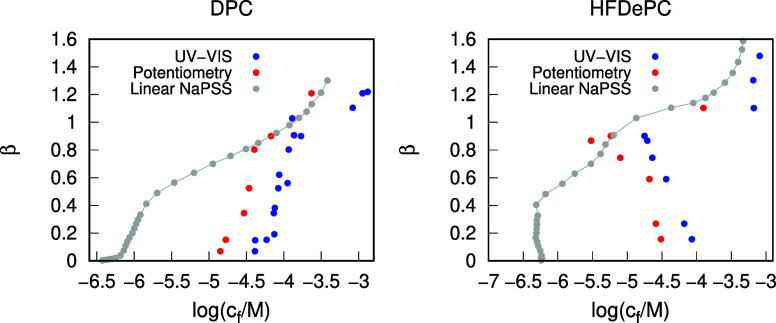
Binding isotherms of DPC and HFDePC to NaPSS hydrogels, determined by UV–vis spectroscopy (blue points) and potentiometry (red points). The gray lines represent the binding isotherms to linear NaPSS determined by potentiometric titration.

It is immediately apparent that, in both the DPC and HFDePC cases, the binding isotherms to NaPSS hydrogels determined potentiometrically and spectroscopically are in good agreement with each other but differ from the binding isotherm to linear NaPSS obtained by potentiometric titration. Reports in the literature suggest that while the binding isotherms of surfactants to linear polyelectrolytes and polyelectrolyte hydrogels may differ in certain aspects (e.g., the β value of coil saturation tends to be slightly higher for hydrogels^55^), they remain qualitatively identical and, in the presence of salt, onset of cooperative binding takes place at nearly the same free surfactant concentration. We can therefore conclude that PSS does, in fact, interfere with the potentiometric measurements of free surfactant concentration. The binding isotherms of DPC and HFDePC to PSS gels presented in Figure 3 are thus “correct” with respect to their position on the *x*-axis. Interestingly, the negative slope displayed by the binding isotherm of HFDePC persists, revealing itself not as an artifact of polyelectrolyte interference but rather as an intrinsic feature of the system. Its origin will be further discussed in section Theoretical Modeling.

The NaPSS hydrogels, equilibrated in surfactant solutions, were studied to gather information on the polyelectrolyte–surfactant aggregates necessary for parametrizing our theoretical models. Examining the masses of hydrogels equilibrated under increasingly more concentrated surfactant solutions, a gradual collapse of the hydrogels can be observed. The progression of the gel collapse can be followed in terms of hydrogel swelling isotherms, presented in Figure 4 (left). Figure 4 (right) also shows examples of NaPSS hydrogel pieces in equilibrium with surfactant solutions at various β values. As reported in literature, the volume phase transition of hydrogels from a fully swollen to a fully collapsed state occurs via the uniform mechanism in case of DPC, whereas in case of HFDePC, a core–shell structure is observed.^6,7,27,56^ The swelling isotherm for HFDePC in Figure 4 has the concave shape characteristic of core–shell phase separated gels.^57^ The effect is explained by the contractive elastic forces in the compact shell acting to reduce the swelling compared to that of homogeneous gels here represented by the DPC case.^58^ The core–shell structure could also be identified by the faint purple coloring on the gel surface when the shell thickness matched the wavelength of light in that part of the spectrum. While this was clearly visible to the naked eye, it has proven to be elusive to capture on camera.

**Figure 4:**
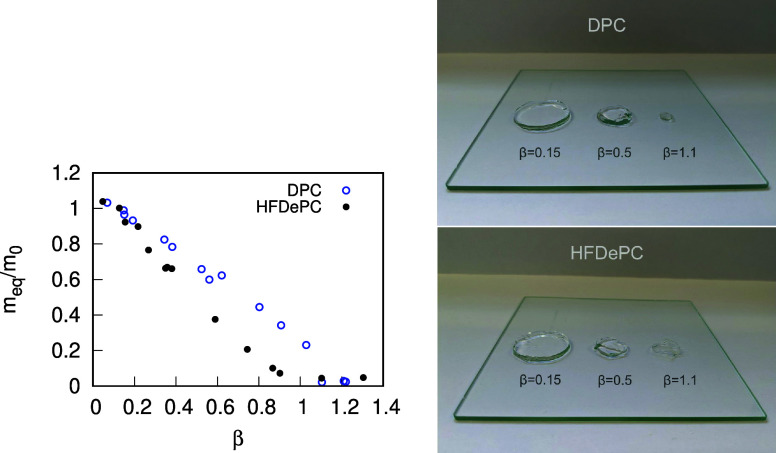
Swelling isotherms of NaPSS hydrogels in equilibrium with DPC or HFDePC solutions (left). Examples of NaPSS hydrogel pieces in equilibrium with DPC (top right) or HFDePC (bottom right) solutions at β values of β = 0.1, β = 0.5, and β = 1.1.

To investigate the micelle shape and distribution within NaPSS hydrogels, selected hydrogel pieces were analyzed using SAXS. At low β values, the micelle shape and size was determined by fitting a form factor to the scattering curves, which are discussed in detail in Section S3 of the Supporting Information. For both DPC and HFDePC, rod-like micelles were formed at moderate β values. At β ≈ 0.6, the micelles measured approximately 12 Å in diameter and 100 or 80 Å in length for DPC and HFDePC, respectively. As β increased, micelle length grew sharply, reaching 1000 Å for DPC and over 3000 Å for HFDePC at β = 0.8. At even higher β values, Bragg peaks appeared in the scattering curves, indicating the cylindrical micelles were packing into a hexagonal lattice within the gel.^6,59,60^ The gradual emergence of these peaks with increasing β is shown in Figure 5. From their positions, the micelle-to-micelle distance in the hydrogel was determined to be approximately 40 Å for both DPC and HFDePC (see Section S3 of the Supporting Information).

**Figure 5:**
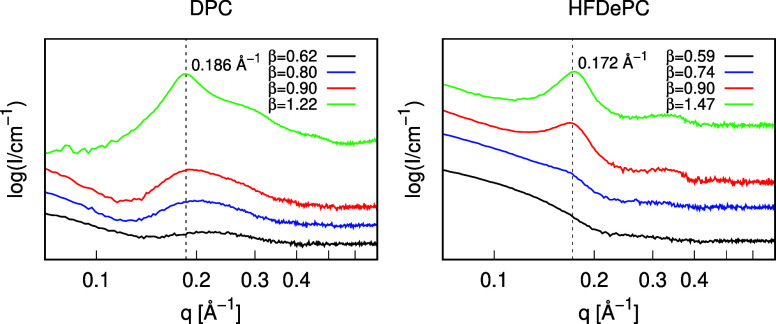
Scattering curves of NaPSS hydrogels equilibrated under DPC or HF solutions at various β values. In both cases, a Bragg peak emerges at high β values (the *q* position of the Bragg peak in each plot is indicated with the dashed black line).

### Theoretical Modeling

Our theoretical treatment focuses exclusively on systems containing a single type of surfactant. This choice stems from the overwhelming complexity of the mixed-surfactant systems, which arises due to a nonuniform distribution of DPC and HFDePC across the PE-surfactant aggregates. The literature suggests that such systems tend to segregate into DPC-rich and HFDePC-rich micelles,^61–63^ or even feature an inhomogeneous distribution of the two surfactants within the same micelle.^62^ These phenomena make the interpretation of experimental results extremely challenging, and further basic knowledge on the micellar structure of the mixed surfactant systems is required before attempting a theoretical treatment of these systems.

The binding isotherms of DPC and HFDePC to NaPSS, calculated using the theoretical models outlined in section Theoretical Models, together with the experimentally determined binding isotherms, are presented in Figure 6.

**Figure 6:**
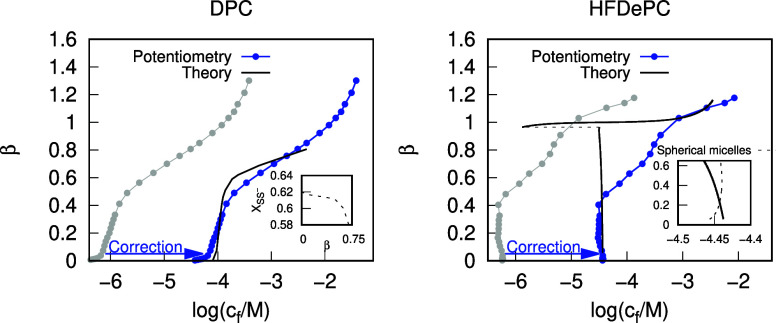
Comparison of theoretically calculated and experimental binding isotherms of DPC (left) and HFDePC (right) to linear NaPSS. The gray lines represent the binding isotherms determined by potentiometric titration, as described in section Surfactant Binding to Linear NaPSS in Solution. The blue lines represent the same isotherms, shifted to the right by the difference between the CAC values determined for linear polyelectrolytes and those determined for NaPSS hydrogels in section Surfactant Binding to NaPSS Hydrogels, to account for polyelectrolyte interference with potentiometric measurements. The theoretical NaPSS-HFDePC binding isotherm corresponds to cylindrical micelle geometry (the nested chart also shows the binding isotherm corresponding to spherical micelle geometry at low β values).

#### Model of the NaPSS-DPC System

Comparison of theoretically calculated and experimental NaPSS-DPC binding isotherms in Figure 6 (left) reveals that the predictions of our model are in semiquantitative agreement with the experimental results. The theoretically calculated isotherm’s shape resembles that measured with the surfactant electrode in solutions of linear NaPSS but the isotherm is positioned at much higher free surfactant concentrations. As already pointed out, the discrepancy can be attributed to the interference of NaPSS with the electrode membrane. However, since cooperative binding to linear polyelectrolytes and the corresponding cross-linked polyelectrolyte gels are expected to start at about the same free surfactant concentration when simple salt is present in the solution,^55^ it is encouraging that the calculated cac is in good agreement with that obtained from UV–vis for the hydrogels. That gives credit to the choice of parameters in the model and shows that the different free-energy contributions are reasonably balanced. Moreover, the model captures the fact that the cooperative binding range is smaller for the linear polyelectrolyte than for the hydrogel. Thus, the theoretical isotherm levels off at β = 0.7, while the UV–vis isotherm reaches its plateau at β = 1. The in-model explanation is that the composition of the mixed DPC-PSS micelles remains relatively constant in a wide range of binding ratios suggesting the existence of an optimal DPC/PSS molar ratio in the micelles, with the mole fraction of SS^–^ segments hovering around *X*_SS^–^_ = 0.6, as is evident in the nested chart in Figure 6 (left). This explanation, emphasizing strong intimate interaction due to combination of electrostatic and hydrophobic attractions, is different from that suggested by Varga et al., who attributed it to weak interactions between the components due to difficulties for the PSS chain to wrap around the micelles.^22^ Our theoretical result aligns with the phase diagram of the related system NaPSS-CTA:SS-water (Sitar et al.^26^), where the component CTA:SS is a charge stoichiometric salt of cetyltrimethylammonium ions and PSS. In the biphasic region where associative phase separation takes place, the dense phase with surfactant/polyion charge ratio close to unity is always in equilibrium with a dilute phase with surfactant/polyion charge ratio of ca. 0.7. This type of phase separation, with conservation of the stoichiometry of the complexes in both phases, is distinctly different from that observed when the interaction between the components is mainly electrostatic, e.g., mixtures of cationic surfactants and sodium polyacrylate.^64^ The authors attributed the behavior to the combined effect of hydrophobic and electrostatic interactions providing a condition for phase separation, that PSS must be associated to a large extent with micelles in both phases in order to minimize the hydrophobic free energy. Clearly, our model calculations, taking into account both hydrophobic and electrostatic interactions, provide a theoretical justification of that view. The same condition explains why the PSS hydrogels underwent a phase transition via a uniform mechanism in the presence of DPC, in agreement with an earlier study of PSS microgels interacting with dodecyltrimethylammonium bromide.^7^

Before ending this section, we mention that it has been recognized that the chemical structure of NaPSS depends on the synthesis method. Balding et al.^65^ reported that the degree of substitution (DS) of commercially available batches of NaPSS from Sigma-Aldrich and Scientific Polymer Products, prepared by sulfonation of polystyrene, was 0.89–0.95, and that physical properties, such as the dependence of radius of gyration on molecular weight, were different from that of NaPSS prepared by controlled radical polymerization of 4-styrenesulfonate monomer. Sen et al.^66^ compared NaPSS prepared by postsulfonation of polystyrene with samples prepared by free radical polymerization and found that both types had DS of 0.95–0.97, indicating that both were essentially fully sulfonated. To explain why the former type had larger osmotic coefficients and surface activity than the latter, they suggested that postsulfonation could result in intramolecular sulfone linkages. However, the sulfone content, which was below the detection limit by the methods used, was appreciated to be <0.1 mol percent. In this work we have used NaPSS from Polysciences with a specified degree of substitution of 1.0. The shape of the DoPC binding isotherms in Figure 2 are very similar to that of dodecyltrimethylammonium bromide in solutions of NaPSS prepared by Vink (DS = 0.98) reported earlier.^48^ Thus, based on the information provided here, there is no reason to believe that DS of the NaPSS used by us would be so low that it would have important effects on the binding pattern of the surfactants. However, the elevated surface activity of NaPSS prepared by postsulfonation of polystyrene may have contributed to the EMF shift in the electrode measurements.

#### Model of the NaPSS-HFDePC System

The theoretical model of the NaPSS-HFDePC system, outlined in section Theoretical Models, can, in principle, be applied to all PE–surfactant systems where interactions between surfactants and the PE chain are predominantly electrostatic. This includes many systems involving conventional hydrocarbon surfactants, such as DPC, and hydrophilic polyelectrolytes like polyacrylate (PA), which have been extensively studied in the past.^3,4,9,51^ However, the binding isotherms of these systems do not exhibit a negative slope in the cooperative binding region. In general, binding isotherms with negative slope are not expected in fully equilibrated systems. In the electrode experiments, the polyelectrolyte solution was titrated with the surfactant solution. This could lead to kinetically stabilized states responsible for the observed effect, e.g., the formation of multichain aggregates that increase in size with increasing surfactant binding or metastable solutions of single chain complexes undergoing conformational transitions induced by the interaction with the surfactant. Below we will argue in favor of the latter mechanism. A possibly related mechanism has recently been used to explain why increased gas pressure can lead to desorption of gas in porous materials. The phenomenon has been observed for responsive matrices that can undergo pressure-induced structural transformations (‘breathing’), such as metal–organic frameworks. However, it appears to require the involvement of metastable phases and is thermodynamically forbidden for rigid adsorbents.^67,68^

Experimental evidence suggests that in dilute aqueous solution, HFDePC tends to form rod-like aggregates.^62^ In contrast, DPC and similar conventional hydrocarbon surfactants form spherical micelles in dilute solutions.^47^ To highlight the difference between the two types of systems, the calculations were performed for both cylindrical and spherical micelle geometries, the former corresponding to the NaPSS-HFDePC system, and the latter to electrostatically interacting systems featuring conventional hydrocarbon surfactants.

Observing the theoretically calculated NaPSS-HFDePC binding isotherm in Figure 6 (right), we see that the model reproduces the negative slope in the cooperative binding region observed in the binding isotherms recorded in solutions of linear NaPSS, as well as for the hydrogels, albeit less pronounced than in the experimental results. Again, the calculated concentration of free surfactant in equilibrium with the complexes at the onset of cooperative binding is in good agreement with the hydrogel data, showing that the model, with the radius of the cylinder micelles and the standard free energy of transfer between water and micelles for the nonpolar part of the surfactant set to physically realistic values (20 Å and −16 *k*_B_ *T*/molecule, respectively) has a good predictive capacity. As shown in the nested chart in Figure 6 (right), this negative slope appears exclusively for cylindrical micelle geometry, underscoring the distinction between polyelectrolyte–surfactant systems with fluorinated surfactants and those with conventional hydrocarbon surfactants, which form spherical micelles. The negative slope in the cylindrical micelle system binding isotherm arises due to the gradual relaxation of the polyelectrolyte coils from an extended conformation as β → 0 to a less strained, more compact configuration as β increases. This relaxation occurs because as the mole fraction surfactants in micelles in the coil increases, the mole fraction of simple positive ions in the coil is in turn reduced. This results in a net decrease in osmotic swelling pressure, allowing the coil to contract to a more ideal conformation. As the coil contracts, X_SS_^–^ in the coil increases, enhancing the electrostatic stabilization of micelles which leads to the observed negative slope. In contrast, for spherical micelles, the reduction of osmotic pressure caused by the expulsion of simple positive ions is counteracted by the increase in osmotic pressure due to the micelle contribution to the entropy of mixing. As a result, the coil is not allowed to relax to the same extent, and the slope of the binding isotherm remains positive throughout the cooperative binding region.

The model calculations provides a mechanism explaining the origin of the negative slope of the binding isotherm. At the same time, it shows that there are collapsed coil states with high degree of binding competing with the swollen states in the negative-slope part. Since that could imply that the lower parts of the binding isotherm represent metastable states rather than true equilibrium states, we let all states with β-values corresponding to stable states represent individual states in a (semi-) grand partition function (see Section S2.2.4 of the SI) and calculated the distribution of HFDePC among the PSS coils for different chemical potentials of the surfactant in the system. Figure 7 shows the resulting ensemble-averaged binding isotherm and the distribution functions for three compositions along the biding isotherm. The binding isotherm is very steep in the cooperative part but the slope is positive for all β. The distribution functions show that a phase transition takes place between micelle-free coils at very low β and collapsed coils with β close to unity. Thus, according to the model, the states in the intermediate binding range with uniform distribution of surfactant among individual coils are metastable states.

**Figure 7:**
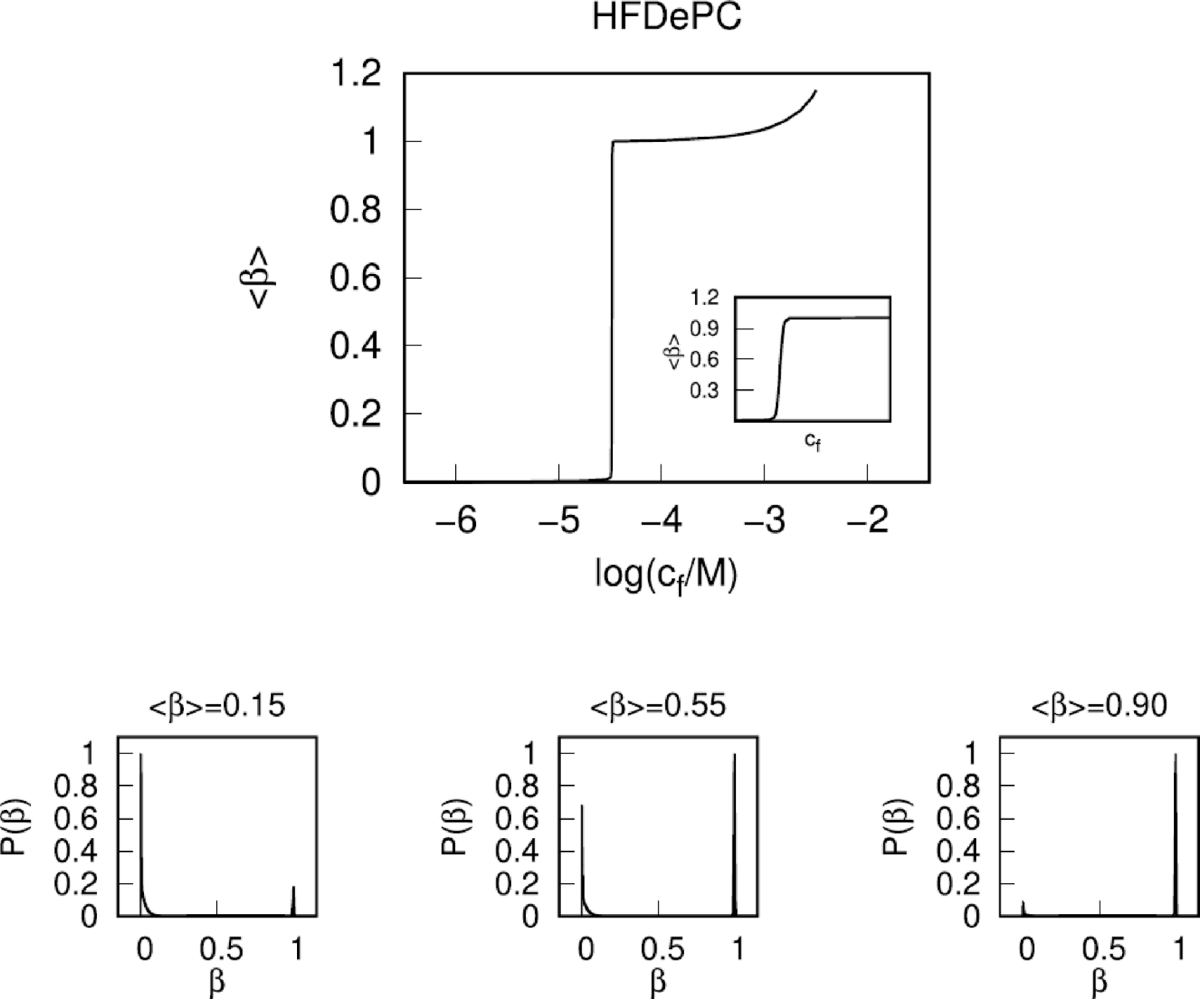
Ensemble-averaged binding isotherm (top) and the normalized distribution functions for three compositions along the biding isotherm (bottom). The nested chart shows a magnified view of the cooperative binding region, highlighting the positive slope of the binding isotherm.

Our overall interpretation of the results is the following: The negative slope of the binding isotherm recorded in the solutions of linear NaPSS is not an artifact of PSS interacting with the membrane. Rather, it reflects the conditions in the swollen coil states, which are metastable states. The metastable states represent kinetically stabilized colloidal states resulting from the titration procedure. They are long-lived because solutions are dilute and because the complexes repel each other (excess negative charge when β < 1). A weak hydrophobic attraction between PSS and the surface of HFDePC micelles may contribute to the stabilization of the metastable state (uniform distribution of surfactant among the PSS chains), which may explain why the binding isotherm tends to “saturate” already at β well below 1, as for DPC. The effect would be a weaker version of that in DPC-PSS; not enough to give a true equilibrium state, but strong enough to create a long-lived metastable solution of net charged complexes. The equilibrium state for concentrations larger than CAC is expected to be phase separated, with a dense phase (precipitate/coacervate) of complexes with β near unity in equilibrium with a dilute NaPSS solution. The negative slope of the binding isotherms for HFDePC-NaPSS gel is most likely an equilibrium phenomenon of different origin than that recorded in solutions of linear NaPSS. Rather, it is a consequence of the core–shell equilibrium as observed earlier with PA gels interacting with cationic surfactants, explained by the gradual relaxation of the elastic energy with increasing β. The effect observed here for HFDePC is larger than expected from the theoretical calculations for spherical gels by Gernandt and Hansson.^58^ The reason could be that the gels are coin-shaped, not spherical. The fact that the latter calculations were made for spherical micelles while HFDePC forms cylinder micelles may also contribute.

## Conclusions

In conclusion, a comparative study of the NaPSS-DPC and NaPSS-HFDePC systems was conducted with the purpose of elucidating the nature of interactions in the fluorinated surfactant-PSS systems.

Binding isotherms of DPC and HFDePC to linear NaPSS in 0.01 M NaCl solutions, determined using potentiometric titration with a surfactant ion-selective electrode, revealed two curious effects. The binding isotherms for both surfactants were positioned at extremely low free concentrations and the slope of the HFDePC isotherm was distinctly negative in the lower binding range and remained unaffected by variations in ionic strength. The former effect has been observed earlier and attributed to a shift of the electrode potential by the binding of PSS to the electrode membrane.^69^ Here we confirmed that this is the case by showing that the effect was absent when the surfactant concentration was measured in PSS-free solutions in equilibrium with NaPSS gels.

The binding isotherm for HFDePC in the gel system likewise displayed a negative slope, even more pronounced than in the solutions of linear NaPSS. However, the gel samples were biphasic of the core–shell type. This was also apparent from the concave shape of the swelling isotherms for HFDePC-PSS gels, characteristic of the core–shell phase separated gels, a behavior distinctly different from the that of the homogeneous DPC-PSS gel samples. Since binding isotherms with negative slope in phase separated gels is known to be a special effect caused by the elastic coupling between the phases, effects not present in liner polyelectrolyte chains, we believe that the negative slope in the binding isotherms measured in the solution is of a different origin.

The results from thermodynamic modeling showed that a binding isotherm with negative slope could be reproduced when HFDePC formed long cylindrically shaped micelles, but not small spherical micelles, a difference explained by the much smaller contribution to the entropy of mixing from cylinder micelles than from spherical micelles. However, thermodynamic ensemble calculations, allowing polymer coils with different amounts of surfactants to coexist, suggest that the intermediate binding states responsible for the negative slope are metastable colloidal states; the equilibrium state is biphasic with micelle-free swollen coils coexisting with collapsed coils with nearly charge stoichiometric amounts of the surfactant and the polyion. Future work is needed to test the metastability hypothesis, e.g., by time-resolved scattering experiments, or studies of mixtures prepared in different ways preferentially by mixing the stoichiometric complex salt HFDeP:PSS with NaPSS and HFDePC, respectively.^26,70^

The thermodynamic modeling of the DPC-PSS interaction suggests that the basic structural unit of the complexes are mixed micelles with surfactant/polyion charge ratio of around 0.6 largely independent of the total binding ratio. The result, which is a combined effect of electrostatic and hydrophobic interactions, explains the comparatively low cooperativity of the binding isotherm and the unusual type of associative phase separation observed in mixtures of NaPSS and conventional cationic surfactants.

Additionally, SAXS was used to gain structural insights into polyelectrolyte–surfactant aggregates within the hydrogels. At moderate β values, both systems formed rod-like micelles, measuring approximately 12 Å in diameter and 100 or 80 Å in length for DPC and HFDePC, respectively, with micelle length increasing sharply as β increased. At β values near coil saturation, Bragg peaks appeared in the scattering curves, indicating the packing of cylindrical micelles into a hexagonal lattice with an intermicelle distance of approximately 40 Å.

## Supplementary Material



## Notes

Experimental procedures for preparation of the SSE and for the NaPSS hydrogel synthesis, detailed description of the theoretical models and the theoretical binding isotherm calculation procedure, and detailed discussion of the SAXS data and additional scattering curves (PDF)
