# Supplementary material for: Interaction of Sodium Polystyrenesulfonate with Fluorinated Ionic Surfactant of Opposite Charge
Source: Langmuir. 2025 Sep 22;41(39):26673–82. doi: 10.1021/acs.langmuir.5c02918 (PMC12509309; doi:10.1021/acs.langmuir.5c02918)
Supplement: Supplementary file 1 [file la5c02918_si_001.pdf]

Supporting Information

Interaction of Sodium Polystyrenesulfonate with Fluorinated Ionic Surfactant of Opposite Charge

Matevž Turk[1], Ksenija Kogej[1], and Per Hansson*[2]

[1]*Department of Physical Chemistry, Faculty of Chemistry and Chemical Technology, University of Ljubljana, Vecna pot 113, 1000 Ljubljana, Slovenia*
[2]*Department of Medicinal Chemistry, Uppsala University, Box 574, SE-75123 Uppsala, Sweden*

S1 Experimental Methods

S1.1 Preparation of the Surfactant Ion-selective Electrode

0.1 M solutions of dodecylpyridinium chloride (DPC) and sodium dodecyl sulfate (SDS) were prepared by dissolving DPC (0.220 g, 1.0 mmol) or SDS (0.288 g, 1.0 mmol) in 10 mL of deionized water. A 2 mL aliquot of the DPC solution (0.2 mmol, 1.0 eq.) was then transferred to a 10 mL beaker, equipped with a stir bar. 2 mL of the SDS solution (0.2 mmol, 1.0 eq.) was added to the DPC solution under constant stirring, which resulted in the formation of the complex $DP^{+}DS^{-}$ as a white precipitate, which was used as a membrane carrier complex. The complex was filtered away, washed 3 times with deionized water and dried under vacuum at 50 °C. The dry membrane carrier complex (0.05 g, 1 wt%) was dissolved in 5 mL of tetrahydrofuran (THF). Polyvinyl chloride (PVC) (1.15 g, 23 wt%) and dioctyl phthalate (3.8 g, 76 wt%) were added, and the mixture was stirred and gently heated until a viscous solution was obtained. The mixture was poured into a petri dish (5 cm diameter). The solvent was evaporated in the fume hood, resulting in the formation of a thin membrane layer on the bottom of the petri dish. The membrane was glued to one end of a plastic tube (1 cm diameter), using a dense solution of PVC in THF as glue. Once dried, 1 mL of the reference DPC solution (0.10125 mM in 0.01 M NaCl) was added to the

*Corresponding author

tube. The other end of the tube was closed with a plastic cap pierced with an Ag|AgCl electrode, which was reaching into the reference solution.

S1.2 NaPSS Hydrogel Synthesis Procedure

The gelling solution was prepared by dissolving styrene sulphonate (2.00 g, 9.7 mmol), *N,N'*-methylenebisacrylamide (0.13 g, 0.84 mmol) and ammonium persulphate (0.01 g, 0.044 mmol) in 10.00 g of water. The solution was deoxygenated using nitrogen gas, after which tetramethylethylenediamine (TEMED) (0.05 g, 0.43 mmol) was added to initiate the polymerization reaction. Ten 1 mL aliquots of the resulting mixture were transferred into separate test tubes and allowed to react. After 7 days, the gels were removed from the test tubes using a pressurized water current and placed in deionized water, which was refreshed six times over the course of 6 hours. Over 24 hours, the gels swelled to 20 times their original size. The hydrogels were stored in a 10^{-5} M solution of sodium azide to prevent fungal growth.

The hydrogels were characterized by determining the monomer concentration within them. A hydrogel piece with an initial wet mass of m_{wet} = 25.592 g was freeze-dried to constant mass (T = -83 °C, P = 232 mT, 1 week). The residual dry mass of the gel was m_{dry} = 0.142 g. Assuming the hydrogel density of ρ_{gel} = 1 $\frac{g}{\text{mL}}$, the monomer concentration in the NaPSS hydrogels was calculated using the formula:

$$c_{m,gel} = \frac{n_{NaSS}}{V_{gel}} = \frac{m_{dry}\rho_{gel}}{M_{NaSS}m_{wet}} = 27\,mM. \tag{1}$$

S2 Theoretical Models

S2.1 Model of the NaPSS-DPC System

In the NaPSS-DPC system, the surfactant ions (DP^+) and the styrene sulfonate segments of the PSS chain (SS^-) form mixed micelles. A micelle consists of a water-free core composed of the hydrocarbon tails of DP^+ and the hydrophobic parts of the SS^- segments, and a charged interface formed by the positively charged pyridinium groups of the surfactant and the negatively charged sulfonate groups of the polyelectrolyte.

S2.1.1 Micelle Characterization

We begin by defining the key quantities and relationships used to characterize the geometry and composition of the mixed micelles.

- Mole fraction of the i-th species in the micelle, X_i, can be calculated as:

$$X_i = \frac{N_i}{N}, \tag{2}$$

  where i can be either DP^+ or SS^-. N_i and N represent the number of i molecules per micelle and the micelle aggregation number, respectively.

- Micelle volume, V_{mic}, can be expressed as follows:

$$V_{mic} = \frac{4\pi}{3} R_{mic}^3 = N_{DP^+}\nu_{DP^+} + N_{SS^-}\nu_{SS^-} = N\left(X_{DP^+}\nu_{DP^+} + X_{SS^-}\nu_{SS^-}\right). \tag{3}$$

In Equation 3, R_{mic} is the micelle radius, while ν_{DP^+} and ν_{SS^-} denote the volumes of the hydrocarbon tail of DP^+ and the hydrophobic part of the SS^- segment, respectively.

- Micelle surface area, A_{mic}, is calculated as:

$$A_{mic} = 4\pi R_{mic}^2. \tag{4}$$

- Area per absolute unit of charge, a, is given by Equation 5:

$$a = \frac{A_{mic}}{|N_{SS^-} - N_{DP^+}|} = \frac{4\pi R_{mic}^2}{N|X_{SS^-} - X_{DP^+}|}. \tag{5}$$

- Area per DP^+ at the micelle surface, a_{DP^+}, can be expressed as:

$$a_{DP^+} = \frac{\frac{4\pi R_{mic}^2}{N} - X_{SS^-}a_{SS^-}}{X_{DP^+}}, \tag{6}$$

where a_{SS^-} represents the area per SS^- at the micelle surface. In our calculations, a_{SS^-} was set to a constant value of 48.7 Å^2, corresponding to the approximate area of a benzene ring.

S2.1.2 Equilibrium Conditions

According to the outline of this model, the NaPSS-DPC system can be imagined as a collection of DP^+ and SS^- species distributing between the water and micelle phases. The mass balance condition for the i-th species can be written as:

$$c_i^{tot} = c_i + N_i c_{mic}, \tag{7}$$

where c_i^{tot} and c_i represent the total and free concentration of i, respectively, and c_{mic} is the concentration of micelles in the system. At equilibrium, the chemical potential of the i-th species in the micelle sub-phase is equal to its chemical potential in the water sub-phase:

$$\mu_i^{mic} = \mu_i^{w}, \tag{8}$$

where the superscripts mic and w represent the micelle and water phases, respectively.

The chemical potential of DP$^+$ in the micelle phase can be expressed as follows:

$$\mu^{mic}_{DP^+} = \mu^{0,mic}_{DP^+} + \gamma a_{DP^+} + \mu^{el,mic}_{DP^+} + k_B T \left(ln \left[X_{DP^+} \right] + X_{SS^-} \right) \\ + \frac{k_B T}{N} \left(ln \left[\frac{c^{tot}_{SS^-}}{c_{SS^-}} - 1 \right] + \frac{c^{tot}_{SS^-}}{c_{SS^-}} - 1 \right). \tag{9}$$

In Equation 9, the first term on the right-hand side, $\mu^{0,mic}_{DP^+}$, is the standard chemical potential of DP$^+$ in the micelle phase. The second and the third term represent the surface energy and the electrostatic energy contributions to the chemical potential, respectively. The final two terms account for the entropy of mixing: the fourth term reflects the entropy arising from the various ways of distributing DP^+ and SS^- species within a micelle, while the fifth term corresponds to the entropy associated with binding the micelles to different sites on the PSS chains [1, 2]. The chemical potential of DP^+ in water can be calculated as:

$$\mu^{w}_{DP^+} = \mu^{0,w}_{DP^+} + k_B T ln \left(\frac{c_{DP^+}}{c_0} \right), \tag{10}$$

where $\mu^{0,w}_{DP^+}$ represents the standard chemical potential of DP^+ in water, and c_0 represents the concentration of water, approximately 55.5 M.

The chemical potential of the SS^- segments in the micelle phase is given by:

$$\mu^{mic}_{SS^-} = \mu^{0,mic}_{SS^-} + \gamma a_{SS^-} + \mu^{el,mic}_{SS^-} + k_B T \left(X_{SS^-} - 1 \right) \\ + \frac{k_B T}{N} \left(ln \left[\frac{c^{tot}_{SS^-}}{c_{SS^-}} - 1 \right] + \frac{c^{tot}_{SS^-}}{c_{SS^-}} - 1 \right). \tag{11}$$

The meaning of each term in Equation 11 corresponds to the meaning of each respective term in Equation 9. The only distinction appears in the fourth term, originating from the reduced degrees of freedom of the SS^- segments due to their linkage within the polymer chain. The chemical potential of the SS^- segments in water is given by:

$$\mu^{w}_{SS^-} = \mu^{0,w}_{SS^-} + \mu^{el,w}_{SS^-}, \tag{12}$$

where $\mu^{0,w}_{SS^-}$ represents the standard chemical potential of an SS^- segment in water, and $\mu^{el,w}_{SS^-}$ represents the contribution of the electric field surrounding a PSS chain to the chemical potential of an SS^- segment in water.

Equations 8 – 12 can be combined into the following relationships:

$$\frac{N}{k_B T} \left(-\Delta \mu^{0}_{SS^-} - \mu^{el,mic}_{SS^-} + \mu^{el,w}_{SS^-} - \gamma a_{SS^-} - k_B T \left(X_{SS^-} - 1 \right) \right) \\ = ln \left[\frac{c^{tot}_{SS^-}}{c_{SS^-}} - 1 \right] + \frac{c^{tot}_{SS^-}}{c_{SS^-}} - 1 \tag{13}$$

$$c_{DP^+} = c_0 e^{\frac{\mu^0_{DP+} + \mu^{el,mic}_{DP+} + \gamma a_{DP+}}{k_B T} + (ln[X_{DP+}] + X_{SS-}) + \frac{1}{N}\left(ln\left[\frac{c^{tot}_{SS-}}{c_{SS-}} - 1\right] + \frac{c^{tot}_{SS-}}{c_{SS-}} - 1\right)}. \tag{14}$$

The terms $\Delta\mu_i^0$ in Equations 13 and 14, represent the difference in the standard chemical potential of the i-th species in the micelle and in the water phase:

$$\Delta\mu_i^0 = \Delta\mu_i^{0,mic} - \Delta\mu_i^{0,w}. \tag{15}$$

S2.1.3 Binding Isotherm Calculation Procedure

Before calculating the binding isotherm, values had to be assigned to the model parameters. The parameters and their corresponding assigned values used in our calculations are presented in Table S1.

Table S1: Parameter values used for the NaPSS-DPC model calculations. The value of ν_{SS^-} was calculated from the density of ethylbenzene. $\Delta\mu^0_{SS^-}$ was used as a fitting parameter.

Parameter	Value
ν_{DP^+}	351 Å^3 [3]
ν_{SS^-}	203 Å^3
a_{SS^-}	48.7 Å^2
N	50 [4]
c_0	55.5 M
$\Delta\mu^0_{DP^+}$	-12 k_BT [5]
$\Delta\mu^0_{SS^-}$	-1 k_BT
γ	0.02 Jm^{-2} [3]

The binding isotherm calculation for the NaPSS-DPC system proceeded in the following steps:

1. A micelle composition is chosen by selecting a value of X_{SS^-}. X_{DP^+} then assumes the value of X_{DP^+} = 1 - X_{SS^-}.

2. For a chosen micelle composition, the micelle radius, R_{mic}, area per absolute unit of charge, a, and area per DP^+ at the micelle surface, a_{DP^+}, are calculated by means of Equations 3 – 6.

3. The electrostatic contributions to the chemical potentials of DP^+ and SS^- segments in micelles, $\mu^{el,mic}_{\mathrm{DP}^+}$ and $\mu^{el,mic}_{SS^-}$, as well as the electrostatic contribution to the chemical potential of SS^- in water, $\mu^{el,w}_{SS^-}$, are determined. $\mu^{el,mic}_{DP^+}$ and $\mu^{el,mic}_{SS^-}$ are calculated by solving the PB equation for spherical symmetry using R_{mic}, a, and a_{DP^+} or a_{SS^-}, respectively, as input parameters [6]. $\mu^{el,w}_{SS^-}$ is calculated by solving the PB equation for cylindrical

symmetry using the linear charge density of the PSS chain and a cylinder radius of $R = 5$ Å as input parameters [6, 7]. All the PB calculations were performed in 10 mM simple salt solutions, matching the concentration used for experimental determination of the binding isotherms to linear NaPSS.

4. The concentrations of SS^- segments and DP^+ in solution, c_{SS^-} and c_{DP^+}, are calculated using Equations 13 and 14, respectively. Finally, surfactant binding ratio, β, is calculated as follows:

$$\beta = \frac{N_{DP^+} c_{mic}}{c_{SS^-}^{tot}} \tag{16}$$

   The value of the total monomer concentration of PSS, $c_{SS^-}^{tot}$, was set to 5×10^{-4} M, matching the concentration used for experimental determination of the binding isotherms to linear NaPSS.

Steps 1 – 4 are repeated for different micelle compositions. The binding isotherm is obtained by plotting the calculated β values against the corresponding free surfactant-ion concentrations on the log scale.

S2.2 Model of the NaPSS-HFDePC System

In the NaPSS-HFDePC system, the interactions between polyelectrolytes and surfactants are predominantly electrostatic. These interactions closely resemble those in systems composed of regular hydrocarbon ionic surfactants and oppositely charged hydrophilic polyelectrolytes, such as NaPA. Such systems can be modelled as a dilute solution of single polyelectrolyte coils, within which surfactant micelles, surfactant unimers, simple salt ions, and water can be enclosed. Each individual coil, along with all the enclosed species, is treated as a small thermodynamic system in equilibrium with the bulk solution, which contains surfactant unimers and simple salt ions but no micelles.

Experimental evidence suggests that in dilute aqueous solution, HFDePC tends to form rod-like aggregates, akin to those observed in the NaPSS hydrogel-HFDePC system by SAXS in Section S3 [8]. In contrast, DPC and similar regular hydrocarbon surfactants form spherical micelles in dilute solutions instead. In the following, we present detailed formulation of our model for electrostatically-interacting polyelectrolyte-surfactant pairs for both spherical and cylindrical geometry of micelles.

S2.2.1 Characterization of a Polyelectrolyte Coil System

A polyelectrolyte coil system is comprised of the following species: water molecules (w), simple monovalent cations ($+$), simple monovalent anions (-), surfactant-ion unimers (S^+), surfactant micelles (mic), and polystyrenesulfonate segments (SS^-). Depending on the polyelectrolyte-surfactant system, the surfactant micelles can either be spherical micelles of a fixed aggregation number N, or cylindrical micelles of infinite length.

We start by defining the mole fraction of the i-th species within the polyelectrolyte coil:

$$X_i = \frac{N_i}{\sum\limits_i N_i}, \tag{17}$$

where N_i is the number of molecules of the i-th species in the polyelectrolyte coil system. In systems containing spherical micelles, N_{mic} can be interpreted simply as the number of micelles within the polyelectrolyte coil. However, for infinite cylindrical micelles, the interpretation of N_{mic} becomes unclear. To address this issue, the micelle content of the polyelectrolyte coil is measured instead in the number of surfactant molecules present in the micelle phase within the polyelectrolyte coil, N_{S+}^{mic}. It is worth noting that this approach can easily be generalized to spherical micelle systems by expressing N_{S+}^{mic} in terms of N_{mic} and the aggregation number N:

$$N_{S+}^{mic} = N_{mic}N. \tag{18}$$

Within a polyelectrolyte coil, surfactant micelles with a radius R_{mic} are located at the centers of cells with a radius $R_{cell} = R_{mic} + L$, where L represents the thickness of the aqueous layer surrounding the micelle [9]. The aqueous layer is populated by water molecules, segments of the polyelectrolyte chain, surfactant unimers, and simple ions. A schematic representation of a cylindrical and a spherical micelle within their respective cells is shown in Figure S1. Since

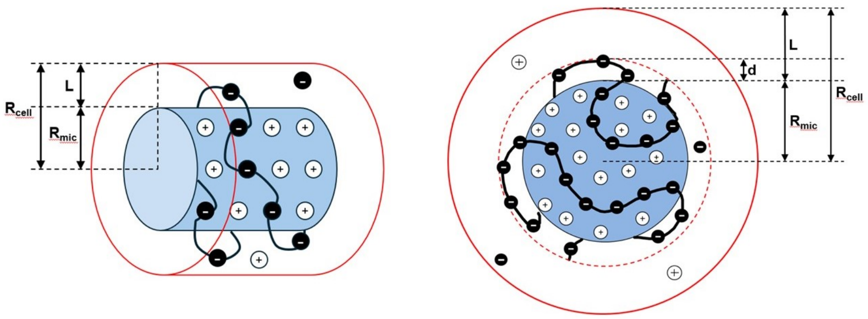

Figure S1: A cylindrical (left) and a spherical (right) micelle within a corresponding cell. The right part of this figure was adapted from the work of Bracic et. al [9] with the permission of the authors.

these cells are assumed to be homogenously distributed within a polyelectrolyte coil, R_{cell} and R_{mic} are directly related to the volume fraction of micelles in a polyelectrolyte coil system, ϕ. For spherical micelles, the following relationship

can be derived:

$$\phi = \left(\frac{R_{mic}}{R_{cell}}\right)^3 = c_{mic}\overline{V_{mic}}. \tag{19}$$

The right-hand side of Equation 19 indicates that ϕ can alternatively be expressed in terms of the molar volume of spherical micelles, $\overline{V_{mic}}$, and the molar concentration of spherical micelles within the coil, c_{mic}, which is given by:

$$c_{mic} = \frac{N_{mic}}{V_p}, \tag{20}$$

where V_p represents the volume of the polyelectrolyte coil. For cylindrical micelles, the volume fraction ϕ can be expressed as:

$$\phi = \left(\frac{R_{mic}}{R_{cell}}\right)^2 = \frac{N_{S+}^{mic}\overline{V_{S+}}}{\overline{V_p}}, \tag{21}$$

where $\overline{V_{S+}}$ represents the molar volume of surfactant unimers.

The free energy expression of a polyelectrolyte coil system can be described as a sum of separable contributions:

$$A = A^{el} + A^{mix} + A^{trans} + A^{surf} + A^{def}. \tag{22}$$

The terms in Equation 22 represent the electrostatic free energy, the free energy of mixing, the free energy associated with transferring fluorocarbon tails from an aqueous environment to the micelle core, the surface free energy, and the configurational free energy of the polyelectrolyte coil, respectively. Each individual term is discussed in more detail below.

- *A^{el}:* For spherical micelles, the electrostatic free energy of a polyelectrolyte coil system was derived by approximating the micelle-containing cells as spherical capacitors [9]:

$$\frac{A^{el}}{k_B T} = N_{mic}\frac{2\pi\sigma^2 R_{mic}^3}{\epsilon\epsilon_0}\left(\frac{d}{R_{mic}+d}\right), \tag{23}$$

  where σ represents the surface charge density of the micelle and $\epsilon\epsilon_0$ is the electric permitivity of the medium. In Equation 23, A^{el} depends on the effective thickness of the polyion layer surrounding the micelles, d, which is a function of L, and can be calculated as follows:

$$d = L_c\left(1 - e^{-\frac{L}{L_c}}\right), \tag{24}$$

  where L_c is a constant that depends on the radius and the charge of the micelles, z_{mic}:

$$L_c = \frac{2R_{mic}}{\sqrt{z_{mic}}}. \tag{25}$$

For cylindrical micelles, the micelle-containing cells were approximated as cylindrical capacitors instead, yielding the following expression:

$$\frac{1}{A_{mic}}\frac{A^{el}}{k_B T} = \frac{\sigma^2 R_{mic}}{2\epsilon\epsilon_0} ln\left(1 + \frac{y_C L}{R_{mic}}\right), \tag{26}$$

where $y_C L$ is given by:

$$y_C L = y_0 R_{mic}\left(1 - e^{-\frac{L}{y_0 R_{mic}}}\right). \tag{27}$$

In Equation 26, the electrostatic free energy is expressed per unit of micelle area, A_{mic}.

- *A^{mix}:* The solution inside the polyelectrolyte coil is assumed to behave ideally with respect to the entropy of mixing of all mobile species. For a spherical micelle system, the mixing free energy term can therefore be expanded as follows:

$$\frac{A^{mix}}{k_B T} = N_w ln(X_w) + N_+ ln(X_+) + N_- ln(X_-) + N_{S^+} ln(X_{S^+}) + N_{mic} ln(X_{mic}) + N_{mic}\left(\frac{4\phi_{HS} - 3\phi_{HS}^3}{(1-\phi_{HS})^2} - 1\right). \tag{28}$$

Here, the polyelectrolyte segments are treated as immobile and contribute to the entropy of mixing only through the calculation of mole fractions. The final term in Equation 28 is the correction for the excluded volume interaction between micelles, derived from the Carnahan-Starling hard-sphere equation of states [10, 11]. The effective volume fraction of the micelle spheres, ϕ_{HS}, is related to the actual volume fraction of micelles, ϕ, and the effective thickness of the polyion layer, d:

$$\frac{\phi_{HS}}{\phi} = \left(\frac{R_{mic} + d}{R_{mic}}\right)^3. \tag{29}$$

In the case of cylindrical micelles, the micellar contribution to the entropy of mixing is neglected. This approximation is justified by the fact that infinite cylindrical micelles, as one-dimensional structures, are confined to distribute in only two spatial dimensions. In contrast, all other species can distribute freely across three spatial dimensions, meaning their contributions to the entropy of mixing significantly outweigh that of the micelles. The mixing free energy expression for cylindrical micelles is therefore analogous to that of the spherical micelles without the final two terms:

$$\frac{A^{mix}}{k_B T} = N_w ln(X_w) + N_+ ln(X_+) + N_- ln(X_-) + N_{S^+} ln(X_{S^+}). \tag{30}$$

- A^{trans}*:* The free energy of transferring the surfactant tails from an aqueous environment to the micelle core can be obtained using the folowing expression:

$$A^{trans} = N_{mic} N \Delta\mu_{tail} = N_{S+}^{mic} \Delta\mu_{tail}, \tag{31}$$

where $\Delta\mu_{\text{tail}}$ is the change in free energy associated with transferring a single surfactant tail from water to the micelle core.

- A^{surf}*:* The surface free energy is calculated as follows:

$$A^{surf} = N_{mic} N(a\gamma) = N_{S+}^{mic}(a\gamma), \tag{32}$$

where a represents the surface area per surfactant molecule on the micelle surface, and γ is the proportionality constant with the units of surface tension.

- A^{def}*:* The configurational free energy of the polyelectrolyte coil is treated analogously to the elastic deformation energy of polyelectrolyte gels [12]:

$$\frac{A^{def}}{k_B T} = -ln\,(\alpha)^3 + \frac{3}{2}\left(\alpha^2 - 1\right) + \left(\frac{\phi}{\phi^*}\right)^{12}. \tag{33}$$

In Equation 33, α measures the deviation of the polyelectrolyte configuration from that of an ideal polymer coil of the same length [13]. It is defined as:

$$\alpha = \left(\frac{V_p}{V_p^{ref}}\right)^{\frac{1}{3}} = \left(\frac{c_p^{ref}}{c_p}\right)^{\frac{1}{3}}, \tag{34}$$

where V_p and V_p^{ref} represent the volumes of the polyelectrolyte coil in the given and reference (ideal polymer coil) states, respectively, and c_p and c_p^{ref} denote the concentrations of monomer segments SS^- within the polymer coil systems in the given and reference states, respectively. The volume V_p is modeled as a sphere with a radius equal to the radius of gyration of the polyelectrolyte coil, *$R(\alpha)$*, in a given state [13]. The final term in Equation 33 represents a Lennard-Jones-like repulsion between micelles within the polyelectrolyte coil, which prevents the volume fraction of micelles in the system from exceeding the critical value, ϕ^*, corresponding to the volume fraction of micelles in a micelle-saturated coil.

There is an underlying assumption in this model that only water molecules and micelles contribute to the volume of the polyelectrolyte coil system, whereas the volume contributions of surfactant unimers, polyelectrolyte segments, and simple salt ions are neglected. The volume of the polyelectrolyte chain system can therefore be expressed as:

$$V_p = N_w \nu_w + N_{mic} \nu_{mic} = N_w \nu_w + N_{S+}^{mic} \nu_{S^+}, \tag{35}$$

where ν_w, ν_{mic}, and ν_{S+} represent the molecular volumes of water molecules, surfactant micelles, and surfactant unimers, respectively.

S2.2.2 Equilibrium Conditions

There are two thermodynamic equilibria present in the NaPSS-HFDePC system:

1. *Equilibrium between the polyelectrolyte coil systems and the bulk solution.* As the polyelectrolyte coil systems are in an equilibrium with the bulk solution, the chemical potentials of each electroneutral component within the polyelectrolyte coil and in the bulk solution can be equated. The chemical potential of the i-th species within the polyelectrolyte coil, μ_i^{coil}, can be obtained by taking the derivative of the free energy expression for the polyelectrolyte coil system, given by Equation 22, with respect to the number of i molecules within the coil, N_i:

$$\mu_i^{coil} = \left(\frac{\partial A}{\partial N_i}\right)_{T,V,N_j}. \tag{36}$$

The chemical potential of the i-th species in the bulk solution, μ_i^{bulk}, can simply be expressed as:

$$\mu_i^{bulk} = k_B T ln\left(\frac{c_i^{bulk}}{c_0}\right), \tag{37}$$

where c_i^{bulk} is the bulk concentration of the i-th species and c_0 is the concentration of water. The equilibrium condition for each species is given below:

- Water:

$$\begin{aligned} \mu_w^{coil} &= \mu_w^{bulk} \\ \mu_w^{el} + \mu_w^{mix} + \mu_w^{def} &= -k_B T \frac{c_+^{bulk} + c_-^{bulk}}{c_0}. \end{aligned} \tag{38}$$

In Equation 38, each term on the left-hand side represents the partial derivative of the corresponding term in Equation 22 with respect to N_w, and the term on the right-hand side represents the first term in the Taylor series expansion of the chemical potential of water in a dilute simple salt solution.

For a spherical micelle system, the electrostatic free energy contribution to the chemical potential of water molecules within the polyelectrolyte coil is given by Equation 39:

$$\frac{\mu_w^{el}}{k_B T} = \frac{\overline{V}_w l_B z_{mic}^2 c_{mic}(1-m)}{6R_{mic}}, \tag{39}$$

where l_B is the Bjerrum length at $T = 298$ K and $\epsilon = 80$ [14], and m is calculated as follows:

$$m = 1 - \frac{e^{\frac{\sqrt{z_{mic}}}{2}(1-\phi^{-1/3})}}{\phi_{HS}^{1/3}}. \tag{40}$$

For a cylindrical micelle system, the electrostatic free energy contribution to the chemical potential of water molecules within the polyelectrolyte coil is given by:

$$\frac{\mu_w^{el}}{k_BT} = \frac{2\pi R_{mic} l_B \nu_w}{a^2(R_{mic}+L)} \frac{e^{-\frac{L}{y_0 R_{mic}}}}{1+y_0\left(1-e^{-\frac{L}{y_0 R_{mic}}}\right)}, \tag{41}$$

where ν_w represents the volume of a single water molecule, and a denotes the area per charge on the micelle surface, which can be expressed as:

$$a = \frac{2\nu_{S^+}}{R_{mic}}, \tag{42}$$

where ν_S^+ represents the volume of a single surfactant molecule.
The entropy of mixing contribution for a spherical micelle system is calculated as:

$$\frac{\mu_w^{mix}}{k_BT} = ln(X_w) + X_{SS^-} - \frac{2\overline{V_w} c_{mic} \phi_{HS} m (2-\phi_{HS})}{(1-\phi_{HS})^3}, \tag{43}$$

whereas for a cylindrical micelle system, the expression simplifies to:

$$\frac{\mu_w^{mix}}{k_BT} = ln(X_w) + X_{SS^-}. \tag{44}$$

The configurational free energy contribution is given by:

$$\frac{\mu_w^{def}}{k_BT} = \frac{\overline{V_w} c_p}{z_p}\left(\left[\frac{c_p^{ref}}{cp}\right]^{2/3} - 1\right) - 12\frac{\overline{V}_w}{V_p}\left(\frac{\phi}{\phi^*}\right)^{12}, \tag{45}$$

where z_p is the charge (and the number of SS^- segments) of a single polyelectrolyte chain.

- Simple monovalent salt:

$$\mu_+^{coil} + \mu_-^{coil} = \mu_+^{bulk} + \mu_-^{bulk} \tag{46}$$

$$\ln(X_+) + X_{SS^-} + \ln(X_-) + X_{SS^-} = \ln\left(\frac{c_+^{bulk}}{c_0}\right) + \ln\left(\frac{c_-^{bulk}}{c_0}\right)$$

- Surfactant salt:

$$\mu_{S^+}^{coil} + \mu_-^{coil} = \mu_{S^+}^{bulk} + \mu_-^{bulk} \tag{47}$$

$$\ln(X_{S^+}) + X_{SS^-} + \ln(X_-) + X_{SS^-} = \ln\left(\frac{c_{S^+}^{bulk}}{c_0}\right) + \ln\left(\frac{c_-^{bulk}}{c_0}\right)$$

Here, it is worth noting again that, in order to simplify the calculations, the contribution to the volume of the polyelectrolyte coil system of surfactant unimers and simple salt ions is neglected. As a result, the partial derivatives of all terms in Equation 22 with respect to the number of these species, with the exception of the entropy of mixing term, are equal to 0.

2. *Equilibrium between the surfactant unimers and surfactant micelles within the polyelectrolyte coil.* Since no micelles are assumed to be present in the bulk solution, the equilibrium between surfactant unimers and surfactant micelles must be considered only within the polyelectrolyte coil systems. By equating the chemical potential of the surfactant molecules in the micelles and those dispersed as unimers, the following expression is obtained:

$$\mu_{S^+,mic}^{coil} = \mu_{S^+}^{coil} \tag{48}$$

$$\mu_{S^+,mic}^{el} + \mu_{S^+,mic}^{mix} + \mu_{S^+,mic}^{trans} + \mu_{S^+,mic}^{surf} + \mu_{S^+,mic}^{def} = k_BT\left(ln(X_{S^+}) + X_{SS^-}\right),$$

where each term on the right-hand side represents the partial derivative of the corresponding term in Equation 22 with respect to $N_{S^+}^{mic}$.

The electrostatic free energy contribution to the chemical potential of spherical micelles within the polyelectrolyte coil is given by:

$$\frac{\mu_{S^+,mic}^{el}}{k_BT} = \frac{l_B z_{mic}}{2R_{mic}}\left(1 - \left[\frac{\phi}{\phi_{HS}}\right]^{1/3}\right) - \frac{\overline{V_{S^+}}}{\overline{V_w}}\left(\frac{1-\phi}{\phi}\right)\frac{\mu_w^{el}}{k_BT}. \tag{49}$$

For cylindrical micelles, the electrostatic free energy contribution is obtained by:

$$\frac{\mu_{S^+,mic}^{el}}{k_BT} = \frac{2\pi R_{mic} l_B}{a} ln\left(1 + y_0\left[1 - e^{-\frac{L}{y_0 R_{mic}}}\right]\right) - \frac{aL(2R_{mic}+L)}{2R_{mic}\nu_w}\frac{\mu_w^{el}}{k_BT}. \tag{50}$$

The entropy of mixing contribution for spherical micelles is calculated as:

$$\frac{\mu_{S^+,mic}^{mix}}{k_BT} = \frac{ln(X_{mic}) + X_{SS^-}}{N} - \frac{1 - (7 - 4m[1-\phi])\phi_{HS} + (10 + 2m[1-\phi])\phi_{HS}^2}{N(1-\phi_{HS})^2}. \tag{51}$$

For cylindrical micelles, the entropy of mixing contribution is equal to 0.

The transfer and surface free energy contributions are obtained by:

$$\frac{\mu_{S^+,mic}^{trans}}{k_BT} + \frac{\mu_{S^+,mic}^{surf}}{k_BT} = \Delta\mu_{tail} + a\gamma. \tag{52}$$

Finally, the configurational free energy contribution is given by:

$$\frac{\mu_{S^+,mic}^{def}}{k_BT} = \frac{\overline{V_{S^+}}c_p}{z_p}\left(\left[\frac{c_p^{ref}}{c_p}\right]^{2/3} - 1\right) + 12\frac{N_w\overline{V}_w}{N_{S^+}V_p}\left(\frac{\phi}{\phi^*}\right)^{12}. \tag{53}$$

In addition to the equilibrium conditions described above, the mass balance and electroneutrality within the polyelectrolyte coil systems must also be satisfied. The expressions for mass balance and electroneutrality are given by Equations 54 and 55, respectively:

$$X_{SS^-} + X_- = NX_{mic} + X_{S^+} + X_+ = \beta^{mic} X_{SS^-} + X_{S^+} + X_+ \tag{54}$$

$$X_{SS^-} + X_{mic} + X_{S^+} + X_+ + X_- + X_w = 1, \tag{55}$$

where β^{mic} denotes the binding ratio contribution of the surfactant molecules in the micelle phase. In the mass balance expression for a cylindrical micelle system, the X_{mic} term can be omitted; as the cylindrical micelles are infinite, their number is negligible compared to the number of other species in the system.

S2.2.3 Binding Isotherm Calculation Procedure

Before calculating the binding isotherm, values had to be assigned to the model parameters. The parameters and their corresponding assigned values used in our calculations are presented in Table S2.

By manipulating the relationships outlined in Sections S2.2.1 and S2.2.2, the problem can be condensed to a system of seven equations (Equations 56 – 62), containing 7 unknowns: X_{SS^-}, c_p, X_{S^+}, X_+, X_-, X_w, and $c_{S^+}^{bulk}$.

$$X_- = -\frac{(1-\beta^{mic})X_{SS^-}}{2} + \sqrt{\left(\frac{(1-\beta^{mic})X_{SS^-}}{2}\right)^2 + \left(\frac{2c_+^{bulk}}{c_0}\right)^2 e^{-2X_{SS^-}}} \tag{56}$$

$$X_w = 1 - (2-\beta^{mic})X_{SS^-} - 2X_- \tag{57}$$

$$c_p = \frac{1}{\beta^{mic}\overline{V_{S^+}} + \frac{X_w}{X_{SS^-}}\overline{V_w}} \tag{58}$$

$$\frac{\mu_w^{mix}}{k_BT} + \frac{\mu_w^{el}}{k_BT} + \frac{\mu_w^{def}}{k_BT} = -\frac{2c_+^{bulk}}{c_0} \tag{59}$$

$$X_{S^+} = e^{\frac{\mu_{S^+,mic}^{el} + \mu_{S^+,mic}^{mix} + \mu_{S^+,mic}^{trans} + \mu_{S^+,mic}^{surf} + \mu_{S^+,mic}^{def}}{k_BT} - X_{SS^-}} \tag{60}$$

$$X_+ = (1-\beta^{mic})X_{SS^-} + X_- - X_{S^+} \tag{61}$$

$$c_{S^+}^{bulk} = \frac{X_{S^+}X_-c_0^2}{c_+^{bulk}} e^{2X_{SS^-}} \tag{62}$$

The binding isotherm calculation for the NaPSS-HFDePC system proceeded in the following steps:

Table S2: Parameter values used for the NaPSS-HFDePC model calculations. The values of z_p and c_p^{ref} can be calculated from the Kuhn segment length (1.54 Å) and the molecular weight of the PSS chains, which was taken to be 70000 Da, matching the molecular weight of the PSS chains used for experimental determination of the binding isotherms to linear NaPSS [13]. The values of ϕ^{max} and R_{mic} for cylindrical micelles were estimated based on the SAXS data presented in Section S3. y_0 was used as a fitting parameter.

Parameter	Value
$c_+^{bulk} = c_-^{bulk}$	10 mM
c_0	55.5 M
$\overline{V_w}$	1.8×10^{-5} m^{3}mol^{-1}
$\overline{V_{S^+}}$	2.48×10^{-4} m^{3}mol^{-1} [15]
ν_w	30 Å^3
ν_{S^+}	412 Å^3
$\Delta\mu_{tail} + a\gamma$	-16 k_BT [16]
z_p	340
c_p^{ref}	2.4 M
l_B	7.14 Å
ϕ^{max}	0.5

Additional parameters for spherical micelles

Parameter	Value
$N = z_{mic}$	40 [17]
R_{mic}	14 Å [17]

Additional parameters for cylindrical micelles

Parameter	Value
R_{mic}	20 Å
y_0	0.1 [18]

1. The value of β^{mic} is chosen.
2. The system of Equations 56 and 59 is solved numerically for X_-, X_w, c_p, and X_{SS^-}.
3. X_{S^+}, X_+, and $c_{S^+}^{bulk}$ are obtained by means of Equations 60 – 62.
4. The degree of binding, β, is calculated from β^{mic} as follows:

$$\beta = \beta^{mic} + \frac{X_{S^+}}{X_{SS^-}} \tag{63}$$

Steps 1 – 4 are repeated for different β values. The binding isotherm is obtained by plotting the calculated β values against the corresponding free surfactant-ion concentrations on the log scale.

The system of Equations 56 – 62 contains multiple solutions in a range of β values around 1. The first set of solutions corresponds to fully swollen, while the second one corresponds to fully collapsed polyelectrolyte coil systems. Conducting the free energy analysis of the two branches of the binding isotherm, it can be observed that the swollen branch is thermodynamically favourable for $\beta < 0.96$, while for $\beta > 0.96$ the collapsed branch is more stable. The swollen and collapsed branches of the binding isotherm with their respective free energies, and the mole fractions of species within the polyelectrolyte coils as a function of β, are presented in Figure S2.

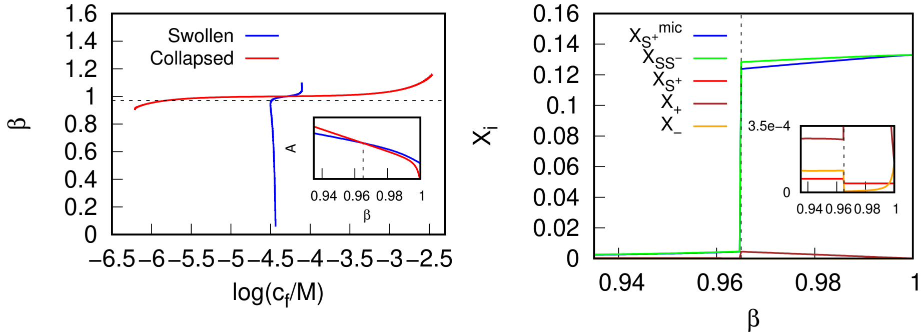

Figure S2: The swollen and collapsed branches of the NaPSS-HFDePC binding isotherm, with their respective free energies (left), and mole fractions of micellized and free surfactant ions (X_{mic} and X_{S^+}), SS$^-$ segments (X_{SS^-}), and simple salt ions (X_+ and X_-) within the polyelectrolyte coil systems of the NaPSS-HFDePC model as a function of β (right). The dashed line indicates the β value at which the transition from the swollen to the collapsed branch of the binding isotherm occurs.

S2.2.4 Ensemble calculations

Let N_i be the number of the species i inside coils with surfactant-to-polyion charge ratio β when the chemical potential of i in the system is μ_i. Then, the fraction of such PSS coils in the system, $P(\beta)$, is equal to:

$$P(\beta) = \frac{e^{\Delta A(\beta)/k_B T}}{Z}, \tag{64}$$

where

$$\Delta A(\beta) = \sum_i \frac{N_i(\beta)\mu_i}{k_B T} - \frac{A(\beta)}{k_B T} \tag{65}$$

$$Z = \int e^{\frac{\Delta A(\beta)}{k_B T}} \, d\beta \tag{66}$$

The ensamble averaged β is then given by:

$$< \beta >= \int P(\beta)\, d\beta \tag{67}$$

S3 SAXS Data

To obtain information about the micelle shape and distribution within the NaPSS hydrogels, selected hydrogel pieces were analyzed by means of SAXS. At low β values, the micelle shape was determined by fitting a form factor to the scattering curves. The values of the form factor parameters, used to fit the scattering curves of hydrogels equilibrated under DPC or HFDePC solutions, are presented in Tables S3 and S4, respectively. The scattering curves, together with the respective fitted form factors, are plotted in Figure S3.

In both cases, a clear trend in the micelle shape can be observed as the β value increases. At higher β values, the core radius decreases and the shell thickness increases. As more surfactant is bound, the length of the micelles also increases, gradually transforming from shorter rod- or ellipsoid-like shapes to long cylinders. At high β values, a Bragg peak emerges in the scattering curves as the cylindrical micelles start packing in a hexagonal lattice within the gel [19–21], as illustrated in Figure S4. The gradual formation of the Bragg peaks as the β value increases can be observed (see Figure 5 in the main article).

Table S3: Parameter values of form factors fitted to SAXS curves of NaPSS hydrogels equilibrated under DPC solutions.

Micelle shape: core-shell cylinder			
β	Core radius [Å]	Cylinder length [Å]	Shell thickness [Å]
0.62	6.22	103.5	15.31
0.80	6.11	992.6	16.73
0.90	5.37	2723.9	19.38

Table S4: Parameter values of form factors fitted to SAXS curves of NaPSS hydrogels equilibrated under HFDePC solutions.

Micelle shape: core-shell ellipsoid			
β	Core equatorial radius [Å]	Core axial radius [Å]	Shell thickness [Å]
0.59	19.58	79.75	34.12
0.74	17.60	3500	39.18

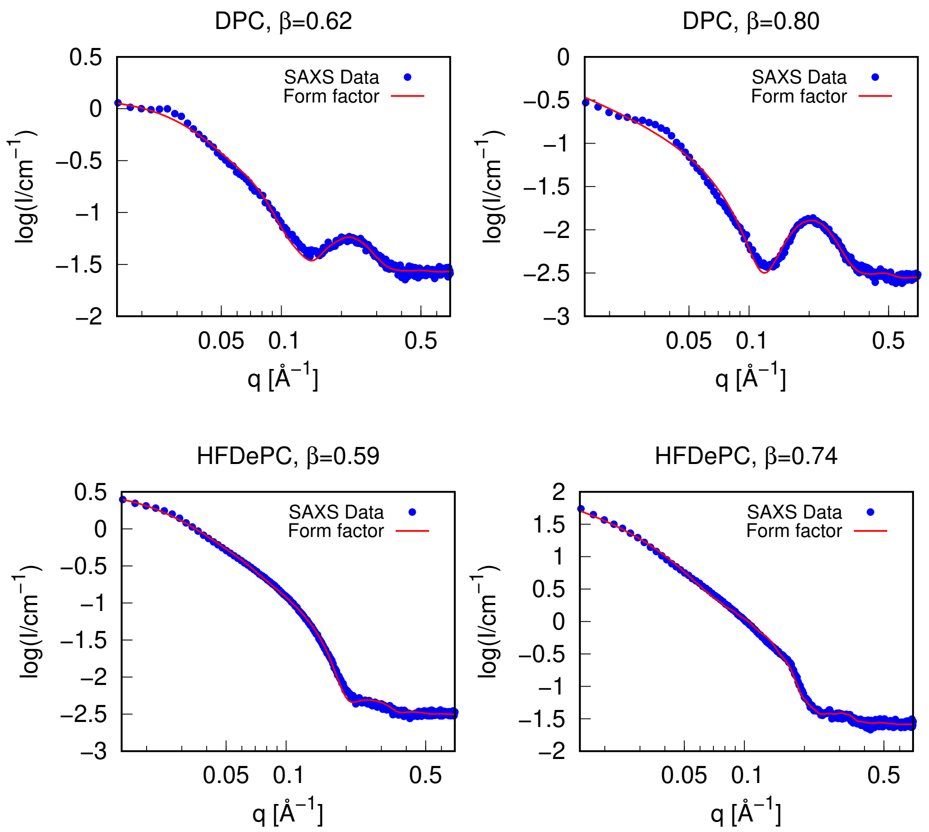

Figure S3: Scattering curves of NaPSS hydrogles equilibrated under DPC (top) or HFDePC (bottom) solutions with the respective fitted form factors.

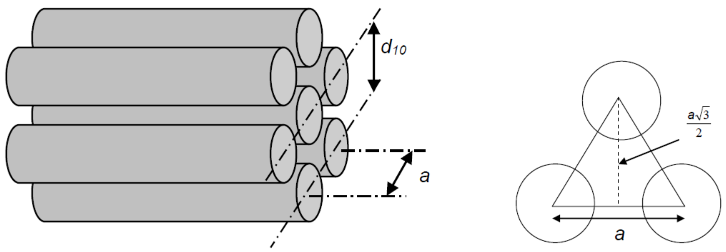

Figure S4: Schematic illustration of cylindrical micelles packed in a hexagonal lattice. d_{10} represents the distance between the scattering planes with Miller indices 1, 0.

From the value of the scattering vector, q, at the position of the Bragg peak, the micelle radius, R, as well as the hexagonal cell dimension, a, (the shortest distance between two adjacent micelles in the lattice, see Figure S4) can be calculated. According to Bragg's law, the q values of the Bragg peaks for cylinders in a hexagonal lattice are given by the following equation:

$$q_{hk} = \frac{4\pi}{a\sqrt{3}}\sqrt{h^2 + k^2 + hk}, \tag{68}$$

where h and k are the Miller indices of the scattering planes. As the first Bragg peak corresponds to the set of scattering planes with Miller indices 1, 0, Equation 68 simplifies to the expression from which a can be readily obtained:

$$q_{hk} = \frac{4\pi}{a\sqrt{3}} \tag{69}$$

The micelle radius can be calculated from the volume fraction of the surfactant in the gel, ϕ_{surf}, which can be expressed as:

$$\phi_{surf} = \frac{\pi R^2/2}{a^2\sqrt{3}/4} = \frac{N_{surf}V_{surf}}{V_{gel}}, \tag{70}$$

where N_{surf}, V_{surf}, and V_{gel} represent the number of the surfactant molecules in the hydrogel, the volume of an individual surfactant molecule, and the volume of the hydrogel, respectively. For a given β value, V_{gel} can be obtained from the swelling isotherm (assuming the hydrogel density of $\rho_{gel} = 1$), and N_{surf} can be calculated from β and the monomer concentration of NaPSS in the hydrogel, determined in Section S1.2. For V_{surf}, experimentally determined values of V_{surf} = 351 Å for DPC [3], and V_{surf} = 656 Å for HFDePC [22], were adopted. The hexagonal cell dimensions and the micelle radii of DPC and HFDePC micelles, calculated from the respective Bragg peaks, are presented in Table S5.

Table S5: Hexagonal cell dimensions and micelle radii of DPC and HFDePC micelles, calculated from the respective Bragg peak positions.

Surfactant	Bragg peak position [Å^{-1}]	Cell dimension a [Å]	Micele radius R [Å]
DPC	0.186	39.06	11.02
HFDePC	0.172	42.18	11.43

References

(1) Hill, T. L., *Thermodynamics of small systems*; Courier Corporation: New York, 1994.

(2) Hansson, P.; Almgren, M. *The Journal of Physical Chemistry B* **2000**, *104*, 1137–1140.

(3) Evans, D. F.; Wennerström, H., *The colloidal domain: where physics, chemistry, biology, and technology meet*; Wiley-Vch New York: 1999.

(4) Andersson, M.; Råsmark, P. J.; Elvingson, C.; Hansson, P. *Langmuir* **2005**, *21*, 3773–3781.

(5) Gernandt, J.; Hansson, P. *The Journal of Physical Chemistry B* **2015**, *119*, 1717–1725.

(6) Jonsson, B.; Wennerstroem, H. *Journal of Physical Chemistry* **1987**, *91*, 338–352.

(7) Iglic, A.; Kulkarni, C. V.; Rappolt, M., *Advances in Planar Lipid Bilayers and Liposomes*; Academic Press: 2012.

(8) Kadi, M.; Hansson, P.; Almgren, M.; Bergström, M.; Garamus, V. M. *Langmuir* **2004**, *20*, 3933–3939.

(9) Bracic, M.; Hansson, P.; Pérez, L.; Zemljic, L. F.; Kogej, K. *Langmuir* **2015**, *31*, 12043–12053.

(10) Carnahan, N. F.; Starling, K. E. *The Journal of chemical physics* **1969**, *51*, 635–636.

(11) Gernandt, J.; Hansson, P. *The Journal of Chemical Physics* **2016**, *144*, 064902.

(12) Hill, T. L., *An introduction to statistical thermodynamics*; Courier Corporation: New York, 1986.

(13) Grosberg, A. Y.; Khokhlov, A. R., *Giant molecules*; World Scientific Publishing: 2010.

(14) Russel, W. B., *Formulation and processing of colloidal dispersions*; Cambridge University Press: Cambridge, 1989.

(15) Krafft, M. P.; Riess, J. G. *Chemical reviews* **2009**, *109*, 1714–1792.

(16) Fendler, J. H. *Advanced Materials* **1996**, *8*, 260–260.

(17) Almgren, M.; Hansson, P.; Mukhtar, E.; Van Stam, J. *Langmuir* **1992**, *8*, 2405–2412.

(18) Hansson, P.; Bysell, H.; Mansson, R.; Malmsten, M. *The Journal of Physical Chemistry B* **2012**, *116*, 10964–10975.

(19) Hansson, P. *Gels* **2020**, *6*, 24.

(20) Leal, C.; Moniri, E.; Pegado, L.; Wennerström, H. *The Journal of Physical Chemistry B* **2007**, *111*, 5999–6005.

(21) Gelbart, W. M.; Bruinsma, R. F.; Pincus, P. A.; Parsegian, V. A. *Physics today* **2000**, *53*, 38–45.

(22) Krafft, M. P.; Riess, J. G. *Chemical reviews* **2009**, *109*, 1714–1792.
